# Increased Circulating Levels of Growth Differentiation Factor 15 in Association with Metabolic Disorders in People Living with HIV Receiving Combined Antiretroviral Therapy

**DOI:** 10.3390/jcm11030549

**Published:** 2022-01-22

**Authors:** Pere Domingo, María Gracia Mateo, Joan Villarroya, Rubén Cereijo, Ferran Torres, Joan C. Domingo, Laura Campderrós, José M. Gallego-Escuredo, María del Mar Gutierrez, Isabel Mur, Noemí Corbacho, Francesc Vidal, Francesc Villarroya, Marta Giralt

**Affiliations:** 1 Infectious Diseases Unit, Institut de Recerca Hospital de la Santa Creu i Sant Pau, 08041 Barcelona, Spain; mmateog@santpau.cat (M.G.M.); jvillarroya@ub.edu (J.V.); rcereijo@santpau.cat (R.C.); jmgallego@ub.edu (J.M.G.-E.); MGutierrezMa@santpau.cat (M.d.M.G.); imur@santpau.cat (I.M.); ncorbacho@santpau.cat (N.C.); 2 Department of Biochemistry and Molecular Biomedicine, Institut de Biomedicina Universitat de Barcelona (IBUB), CIBER Fisiopatología de la Obesidad y Nutrición (CIBEROBN), 08028 Barcelona, Spain; jcdomingo@ub.edu (J.C.D.); lcampderros@ub.edu (L.C.); fvillarroya@ub.edu (F.V.); mgiralt@ub.edu (M.G.); 3 Biostatistics and Data Management Core Facility, IDIBAPS, Hospital Clinic Barcelona, 08036 Barcelona, Spain; Ferran.Torres@uab.cat; 4 Biostatistics Unit, Faculty of Medicine, Universitat Autònoma de Barcelona, 08193 Barcelona, Spain; 5 Infectious Diseases Unit, Department of Internal Medicine, Hospital Universitari Joan XXIII, IISPV, Universitat Rovira i Virgili, 43003 Tarragona, Spain; fvidal@comt.es

**Keywords:** GDF15, cardiovascular risk, Framingham, D:A:D, insulin resistance, metabolic syndrome, HALS

## Abstract

Objective: People living with HIV (PLWH) have an increased cardiovascular risk (CVR) owing to dyslipidemia, insulin resistance, metabolic syndrome, and HIV/combination antiretroviral therapy (cART)-associated lipodystrophy (HALS). Atherosclerosis and inflammation are related to growth differentiation factor-15 (GDF15). The relationship between metabolic disturbances, HALS, and CVR with GDF15 in PLWH is not known. Research design and methods: Circulating GDF15 levels in 152 PLWH (with HALS = 60, without HALS = 43, cART-naïve = 49) and 34 healthy controls were assessed in a cross-sectional study. Correlations with lipids, glucose homeostasis, fat distribution, and CVR were explored. Results: PLWH had increased circulating GDF15 levels relative to controls. The increase was the largest in cART-treated PLWH. Age, homeostatic model assessment of insulin resistance 1 (HOMA1-IR), HALS, dyslipidemia, C-reactive protein, and CVR estimated with the Framingham score correlated with GDF15 levels. The GDF15-Framingham correlation was lost after age adjustment. No correlation was found between GDF15 and the D:A:D Data Collection on Adverse Effects of Anti-HIV Drugs (D:A:D) score estimated CVR. CVR independent predictors were patient group (naïve, HALS−, and HALS+) and cumulated protease inhibitor or nucleoside reverse transcriptase inhibitor exposure. Conclusions: PLWH, especially when cART-treated, has increased GDF15 levels—this increase is associated with dyslipidemia, insulin resistance, metabolic syndrome, HALS, and inflammation-related parameters. GDF15 is unassociated with CVR when age-adjusted.

## 1. Introduction

Combination antiretroviral therapy (cART) has forever changed the landscape for people living with HIV (PLWH) [1]. Consistently, mortality rates have decreased, and a significant change in the PLWH morbidity pattern has occurred. Morbidity associated with acquired immunodeficiency syndrome (AIDS)-defining conditions have steadily decreased over time, whereas aging-associated co-morbid conditions have gained prominence. Among these conditions, metabolic and fat distribution disturbances, together with increased CVR, stand out [2].

A scenario of atherogenic dyslipidemia is depicted in cART-treated PLWH owing to lipid disturbances and insulin resistance, which may translate into increased CVR [3]. Besides, adipose tissue abnormalities link with an even worse lipid profile [4]. Full-blown HIV/cART-associated lipodystrophy (HALS) is often associated with the metabolic syndrome, a well-known cluster of risk factors for cardiovascular disease (CVD) [5,6]. The incidence of myocardial infarction in PLWH is increased by 26% relative to uninfected controls in longitudinal studies [7,8]. Not surprisingly, endothelial dysfunction and subclinical atherosclerosis are more frequent in PLWH than in uninfected subjects [9].

Growth differentiation factor-15 (GDF15) or macrophage inhibitory cytokine-1, a member of the transforming growth factor-b superfamily, is related to inflammation, chronic vascular diseases, cancer, ischemia, and atherosclerosis, behaving as a lesion-induced factor [10,11]. GDF-15 levels are increased in human obesity, diabetes, and genetic lipodystrophy [12,13] but also in patients with anorexia nervosa [14] or after feeding restriction [15], and current views postulate a role for GDF15 in connecting somatic distress signals to the central control of energy balance [16]. Moreover, GDF-15 functions as a cardiovascular risk and outcome marker, and there are indications that it may participate directly in the development of the atherosclerotic process [11]. Thus, GDF15 circulating levels strongly associate with mortality across a spectrum of CVD states, including chest pain, acute coronary syndromes, stable coronary heart disease (CHD), and heart failure [17,18].

PLWH are prone to accelerated atherosclerosis, metabolic syndrome and increased risk of cardiovascular events, especially when developing HALS. Because GDF15 is suggested as a marker of atherosclerotic burden and metabolic dysregulation, our working hypothesis was that circulating GDF15 levels could be associated with CVR, metabolic alterations and HALS in PLWH.

## 2. Patients and Methods

### 2.1. Subjects

This is a retrospective cross-sectional study (see Supplementary Figure S1), the primary outcome of which was to establish circulating levels of GDF15 in PLWH with and without lipodystrophy, untreated HIV-infected patients and healthy controls for comparison, and to relate it with indications of CVD. All patients were recruited between June 2012 and June 2015. All patients were recruited through the same clinic at the Hospital de la Santa Creu i Sant Pau, which attended a population of 1810 HIV-infected patients on active follow-up and were consecutive patients with an established diagnosis of HIV infection. Patients were eligible whether they had HIV/combination antiretroviral therapy (cART)-associated lipodystrophy syndrome (HALS), which was assessed as described in [19] and based mostly on the scales defined by Lichtenstein et al. [20], and whether they were on cART. Cumulative exposure to each antiretroviral drug was quantified in months after the onset of treatment until the moment of data collection or termination of treatment with each specific drug, as described previously [21,22]. Subjects hospitalized or who had a frank cognitive impairment such as delirium or dementia upon enrollment were not eligible. Patients with opportunistic infections, acute hepatitis, liver insufficiency, chronic congestive heart failure, neoplasms, or fever of undetermined origin were excluded from the study, as were individuals taking anti-inflammatory or immunomodulatory drugs. The diagnosis of AIDS was based on the 1993 revised case definition of the Centers for Disease Control and Prevention (CDC) [23]. Controls were recruited among hospital personnel, including physicians, nurses, and auxiliary personnel. They were negative for HIV infection, between 35 and 50 years of age and with a proportion of males of about 70%, to fit the age and gender proportion average of the PLWH groups. The exclusion criteria were the same as above for PLWH. Written informed consent was obtained at study entry from all participants. The study was approved by the Ethics Committee of the Hospital de la Santa Creu i Sant Pau, and it was performed following relevant guidelines/regulations.

### 2.2. Biochemistry Laboratory Measurements

Blood was obtained in the morning, between 8 a.m. and 9 a.m. local time, after a 12 h overnight fast and at least 15 min after placing a peripheral intravenous catheter. Serum samples were split stored as frozen aliquots and when samples recruitment had been completed, a set was delivered to the core laboratory at Hospital Santa Creu i Sant Pau, Barcelona (Spain) for biochemistry laboratory measurements and another to set to the Department of Biochemistry and Molecular Biomedicine of the University of Barcelona (Spain) where GDF15, cytokine and fatty acids were measured. All samples were processed at the same time for analyte quantification. As previously described, all lipid measurements were performed using a Hitachi 911 system (Roche Diagnostic Systems, Basel, Switzerland) [24]. The diagnosis of diabetes mellitus was based on the criteria of the American Diabetes Association [25]. As previously described, insulin resistance was estimated by the homeostasis model assessment method 1 (HOMA1-IR) [26]. The estimated glomerular filtration rate (eGFR) was determined with the CKD-EPI algorithm [27].

### 2.3. Body Composition Measurements, the Definition of HALS, Metabolic Syndrome, and Cardiovascular Risk 

Weight, body mass index (BMI), and waist circumference were measured as described elsewhere [24]. Whole-body DXA scans (Hologic QDR-4500A Hologic, Inc., 590 Lincoln St, Waltham, MA 02154, USA) were conducted by a cART-blinded single operator [24]. HALS was defined as previously described (see Supplementary Methods). Metabolic syndrome was defined according to the stated guidelines [28]. Cardiovascular risk at ten years was assessed with the Framingham risk score [29], and at five years, with the Data Collection on Adverse Effects of Anti-HIV Drugs (D:A:D) equation risk score for PLWH [30].

### 2.4. GDF15, Cytokine, and Fatty Acid Circulating Levels

Serum GDF15 levels were determined in duplicate for each sample using an ELISA specific for human GDF15 (R&D Systems, McKinley Place NE, MN, USA) (Median for the whole cohort: 747.4 pg/mL; IQR: 1275.8; intra- and inter-assay coefficients of variation: 2.2% and 4.7%, respectively) that detects specific human GDF15. Serum levels of IL-6, IL-8, TNF-a, monocyte chemoattractant protein 1 (MCP-1) and leptin, were detected in duplicate for each sample using an antibody-linked, fluorescently labeled microsphere bead-based multiplex analysis system (Linco Research/Millipore, Billerica, MA, USA) and quantified using Luminex 100ISv2 equipment (median for whole cohort: IL-6: 128 pg/mL, IQR: 73.0–247.5; IL-8: 435 pg/mL, IQR: 312.0–659.5; TNF-a: 386 pg/mL, IQR: 264.3–602.8; MCP-1: 197 pg/mL, IQR: 163.0–242.5; leptin: 1100 pg/mL; IQR: 26.4–7159; intra- and inter-assay coefficients of variation: IL-6: 2% and 10%; IL-8: 3% and 14%; TNF-a: 3% and 19%; MCP-1: 2% and 11%; and leptin 5.1% and 12%, respectively). Serum adiponectin levels were measured, also in duplicate, using a specific ELISA kit for human adiponectin (Millipore, Billerica, MA, USA) (median for the whole cohort: 1.9 μg/mL; IQR: 1.2–5.0; intra- and inter-assay coefficients of variation: 7.4% and 8.4%, respectively). The serum composition of fatty acids was determined using the method of Lepage and Roy as previously described [31].

### 2.5. Statistical Analyses

This was a pilot study, and because the prevalence of disturbances of GDF15 levels among PLWH is unknown, we planned to study 200 subjects. HIV-infected patients will be 150 antiretroviral-experienced and 50 uninfected controls. With an overall sample of 200 subjects and an estimated loss of 10%, a difference of ≥20% in the primary variable (GDF15 levels) can be detected, which seems clinically meaningful, assuming a variability of about 30%, a statistical power of >90% and a two-sided type I error of 5%. The study would still have an 80% or 90% statistical power for effect sizes as small as 0.46 or 0.54, respectively. Moreover, 200 subjects let us explore predictive factors in a multivariable logistic model with a sensible number of variables. Data are expressed as frequencies and percent for categorical and median (25th–75th percentiles, interquartile range-IQR-) for continuous variables, or as specified otherwise. When needed, variables were normalized by logarithmic conversion for statistical analysis. The Fisher’s exact test was used for categorical variables and nonparametric tests (Mann–Whitney test for two groups or Kruskal–Wallis for more than two groups) for continuous variables. Correlations were assessed using the Pearson method, and when adjusted by age or comorbidities, assessed using partial correlations. Stepwise ordinal logistic regression analysis was used to examine the association with the Framingham (categorized into three risk levels: <10, 10–20, >20) and D:A:D (categorized into tertiles) CVR scores. The variables selected to enter stepwise regression were those that correlated significantly with CVR. All analyses were performed using SAS 9.4 software (SAS Institute Inc., Cary, NC, USA) or Statistical Package for Social Sciences version 21.0 (SPSS, Chicago, IL), and a level of significance was established at the two-sided 5% level, except for the multivariate logistic model where the 10% level was predefined.

## 3. Results

### 3.1. Population Studied

One hundred and fifty-two PLWH (49 untreated patients, 43 cART-treated patients without HALS, and 60 cART-treated patients with HALS) and 34 healthy controls were studied. Their mean ages were 42.4 ± 9.7 and 40.8 ± 3.1 years, respectively (*p* = 0.1338). Demographic, clinical data and antiretroviral exposure are shown in Table 1. Mean duration of HIV infection was 10.2 ± 6.3 years (median: 10.5 [IQR: 5.0–15.0 years]), and 46 patients (30.2%) had had an AIDS-defining condition. Forty-seven patients were co-infected with hepatitis C virus (30.9%), whereas 10 (6.5%) had chronic hepatitis B virus infection. Twelve patients (7.9%) had diabetes. Six patients (3.9%) had an eGFR <60 mL/min/1.73 m^2^. There were 49 naïve patients (32.2%), 43 without HALS (28.2%), and 60 with HALS (39.5%). Anthropometric, CVR factors, metabolic, fat, viral, and therapeutic data, together with serum cytokine levels, are shown in Table 2.

### 3.2. Antiretroviral Drug Exposure and Immuno-Virological Status

Among treated patients, 83 (80.6%) had undetectable viral load at study entry. The other 20 treated patients presented a median detectable viral load of 2.4 log_10_ copies/mL (IQR: 2.1–3.0 log_10_ copies/mL). The mean CD4 count was 608 ± 345 cells/mm^3^ (median: 544 [IQR: 352–813] cells/mm^3^). Nadir CD4 cell count was <100 cells/mm^3^ in 41 patients (26.9%). There was a strong correlation between cumulated exposure to nucleoside reverse transcriptase inhibitors (NRTI) (r = 0.542, *p* < 0.001), especially thymidine analogues (r = 0.385, *p* < 0.001), and abacavir (r = 0.344, *p* < 0.001) with GDF15 circulating levels, even when age-adjusted. Cumulated exposure to protease inhibitors (PI) (r = 0.382, *p* < 0.001) also correlated with GDF15 levels (Supplementary Table S1). The association of integrase inhibitors with GDF-15 could not be assessed because none of our patients were on integrase inhibitor-based regimen.

### 3.3. GDF15 Circulating Levels in Controls and PLWH

The median serum GDF15 levels for the whole cohort were 2.86 (IQR: 2.56–3.21) log_10_ pg/mL. There was correlation between GDF15 levels and age (r = 0.453, *p* < 0.001). There were no differences in GDF15 levels by gender. GDF15 levels were higher in patients than in controls (2.99 [IQR 2.74–3.32] vs. 2.45 [IQR 2.32–2.58] log_10_ pg/mL, respectively) (*p* < 0.0001). GDF15 levels were higher than 3.08 (1200 ng/L) and 3.26 log_10_ pg/mL (1800 ng/L) in 35% and 24.5% of patients, respectively, compared with 15.4% and 7.7% of controls (*p* = 0.06 for both). GDF15 levels were not significantly different in smokers, HCV, or HBV co-infected patients, neither regarding their HIV detectability or CD4 count status, but were higher in diabetic (*p* = 0.001) and hypertensive patients (*p* = 0.043) (Supplementary Table S2).

### 3.4. GDF15 Circulating Levels, Fat Mass, HALS, and Metabolic Syndrome

**Figure 1:**
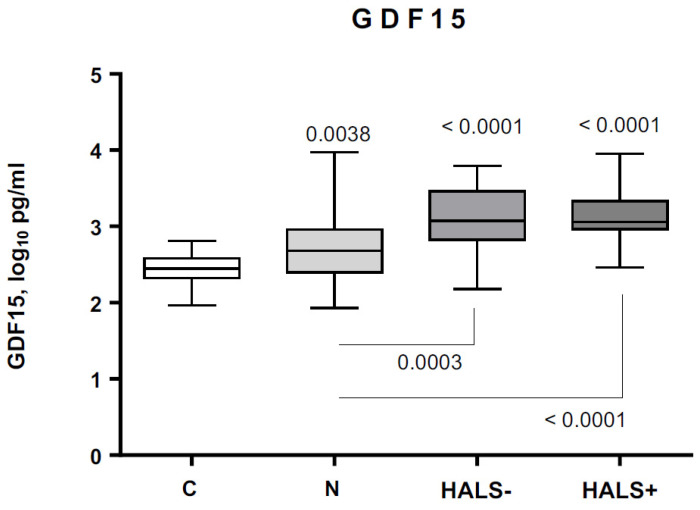
Circulating levels of GDF15 in controls and PLWH with or without HIV/cART-associated lipodystrophy (HALS) GDF15 = Growth differentiation factor 15; C = controls N = Naive HIV-infected patients HALS− = HIV-infected patients without HIV/cART-associated lipodystrophy HALS+ = HIV-infected patients with HIV/cART-associated lipodystrophy.

The correlation between anthropometric, metabolic, circulating and infection/treatment-related parameters and GDF15 in PLWH is shown in Table 3. Neither BMI nor waist-to-hip ratio were associated with GDF15 levels (Supplementary Table S3). GDF15 levels were higher in naïve patients relative to controls and further increased in cART-treated patients, but no changes were found according to HALS development (Figure 1). Whole-body fat, trunk fat, and appendicular fat mass did not correlate with GDF15 levels when controlling by metabolic syndrome and diabetes (Supplementary Table S3). Coherently, there were 28 patients (18.4%) who met the diagnostic criteria for metabolic syndrome. Their respective GDF15 levels were 3.06 [2.69–3.46] and 2.82 [2.54–3.13] log_10_ pg/mL for those with or without metabolic syndrome (*p* = 0.044).

### 3.5. GDF15 Circulating Levels, Lipids, Glucose Homeostasis, and Inflammation

GDF15 levels correlated with LDL cholesterol, PUFA, MUFA, FGF21, and insulin resistance, even after age adjustment, whereas they did not correlate with triglycerides, HDL cholesterol, or non-HDL cholesterol (Table 3 and Supplementary Table S3). GDF15 levels correlated significantly with TNFα, interleukin-6, and IL-8, as well as with C reactive protein, indicating a positive association between GDF15 and biomarkers of inflammation. These correlations were maintained after age adjustment (Table 3).

### 3.6. GDF15 Circulating Levels and Cardiovascular Risk

Because alterations in GDF15 circulating levels have been associated with increased atherosclerotic burden as mentioned before [11,17], we next proceeded to analyze the possible connection between GDF14, CVR as measured by the Framingham and D:A:D scores and other variables of interest in our PLWH cohort. The median CVR at 10 years according to Framingham risk score was 3% (1–4%) for controls and 7% (4–14%) for PLWH (*p* < 0.0001). Among PLWH, CVR strata were <10% in 96 patients (63.1%), 10–19% in 34 (22.4%) and >20% in 22 (14.5%). Serum GDF15 levels correlated with Framingham score risk, but this correlation was lost after adjustment for age (Table 3). The median five-year CVR in PLWH according to D:A:D equation was 3.04% [1.23–5.48]. There was no correlation between D:A:D score and GDF15 circulating levels (r = 0.05, *p* = 0.5984), even without age adjustment. Correlations with CVR scores are shown in Table 4 and Supplementary Table S4. There were 11 patients with prior CVD diagnosed by vascular clinicians—CHD in 9 of them, peripheral arterial disease in 1 (assessed by ankle-brachial index determination and Doppler ultrasonography), and cerebrovascular disease in 1. GDF15 levels were 3.34 [IQR 2.93–3.47] log_10_ pg/mL in patients with prior CVD, whereas they were 2.82 [IQR 2.55–3.15] log_10_ pg/mL in those without (*p* = 0.041).

### 3.7. Subgroup Analyses

All significant correlations with glucose homeostasis and triglycerides disappeared after removal of patients with diabetes mellitus. After removal of patients with previous CVD, GDF15 levels remained correlated to markers of insulin resistance (insulin, r = 0.302, *p* = 0.001; HOMA1-IR, r = 0.234, *p* = 0.010; FGF21, r = 0.216, *p* = 0.011), duration of infection (r = 0.182, *p* = 0.042), cumulated NRTI exposure (thymidine NRTI: r = 0.181, *p* = 0.038), non-nucleoside reverse transcriptase inhibitors (NNRTI) (r = 0.257, *p* = 0.004), and lopinavir/ritonavir (LPV/r) (r = 0.185, *p* = 0.042). On removing patients with eGFR <60 mL/min/1.73 m^2^, glucose homeostasis markers remained correlated to GDF15 levels (insulin: r= 0.276, *p* = 0.002; HOMA1-IR r = 0.240, *p* = 0.008, FGF21: r = 0.187, *p* = 0.041) as well as cumulated NRTI exposure (r = 0.233, *p* = 0.010), NNRTI = 0.212, *p*= 0.019, and LPV/r (r= 0.212, *p* = 0.019)

### 3.8. Independent Predictors of Cardiovascular Risk

An ordinal logistic regression analysis was performed in order to determine which variables could act as independent predictors of CVD risk. Both risk scores (Framingham and D:A:D) were used as the dependent variables, and duration of infection, patient group, nadir CD4 cell count, current HIV-RNA, BMI, systolic BP, diastolic BP, trunk/appendicular fat ratio, triglycerides, total cholesterol/HDL ratio, cumulative NRTI and PI exposure, AIDS, C reactive protein, and GDF15 levels were used as independent variables. CVR was categorized using the standard cut-off risk levels (i.e., <10% for low risk, 10–20% for intermediate-risk, and >20% for high risk). The univariate analysis, conducted for all variables, identified the same variables as those statistically significant in analyzing correlations with CVR (Table 4), except triglycerides and total cholesterol/HDL ratio, thus further confirming the results shown in Table 4 and associating the significant variables to specific cut-off risk levels for CVD. The stepwise approach identified the following independent predictors: patient group (control, naïve, HALS− and HALS+, *p* = 0.009) and PI total exposure (*p* = 0.082). The analysis using the five-year D:A:D CVR leads to similar results for the risk correlations and for identifying independent predictors: group of patients (naïve, HALS− and HALS+, *p* = 0.034), NRTI total exposure (*p* = 0.042) and PI total exposure (*p* = 0.031).

## 4. Discussion

Our study shows that PLWH has increased GDF15 levels compared with uninfected controls, in agreement with previous reports [32,33,34]. Increased GDF15 levels in PLWH parallel abnormalities in fat distribution, consistent with the reported association of GDF15 with metabolic and adipose abnormalities and glucose homeostasis disturbances in the non-infected population [35]. All these disturbances are prevalent in PLWH, who develop an excess of aging-related comorbidities in the setting of otherwise well-controlled infection [2]. Insulin resistance, kidney dysfunction, and dyslipidemia are, in turn, risk factors for CVD [36], and CVR is increased in PLWH, as our study exemplifies. GDF15 circulating levels are associated with atherosclerotic burden in uninfected people and varied populations [17,18]. Notwithstanding that, we could not find an association between GDF15 circulating levels and estimated CVR in PLWH, either with Framingham or D:A:D CVR scores. However, like other studies [4,8], the development of fat redistribution and the cumulated PI exposure increased CVR.

GDF15 concentrations have been associated with total and cardiovascular mortality and inconsistently with nonfatal myocardial infarction [18,37], and GDF15 has been proposed as a biomarker for cardiovascular events and all-cause mortality in the general population and PLWH [32,35]. In CHD studies, the threshold of 1200 ng/L (3.08 log_10_ pg/mL) was derived as the upper limit of normal (90th percentile) in healthy European men and women, representing, at the same time, the lower tertile boundary in patients presenting with acute coronary syndromes [17]. The upper tertile boundary in this high-risk population was 1800 ng/L (3.26 log_10_ pg/mL), which was clearly associated with increased mortality [38,39,40]. Among our patients, GDF15 concentrations were above the cutoffs of 3.08 and 3.26 log_10_ pg/mL in a third and a quarter, respectively, suggesting that a substantial proportion of patients in our cohort is at high risk for a cardiovascular event. Despite that, we could not correlate GDF15 levels with estimated CVR by Framingham and D:A:D score when adjusted by age.

High GDF15 circulating levels have been observed in patients with type II diabetes mellitus or obesity related to impaired glucose control [41]. This situation was mimicked in our patients with solid correlations with glucose homeostasis parameters, especially FGF21, fasting insulin, and HOMA1-IR, the latter two reflecting whole-body insulin resistance. However, we did not find a correlation of GDF15 levels either with general anthropometric measurements or with altered fat distribution as evidenced by appendicular and trunk fat, an indirect marker of visceral fat excess, which may be nonetheless related to the absence of obesity among our patients. Even though GDF15 has been linked to anorectic effects [42], no such effects were seen in our patients, and consistently no correlation between BMI and GDF15 circulating levels was found.

Even though the cellular source of GDF15 in diabetic and obese patients has not been established, it is known that GDF15 is expressed in and secreted from human adipocytes and possibly by macrophages infiltrating adipose tissues [43], and it seems that GDF15 can be a marker of inflammation and insulin resistance in adipose tissue [44]. Adipose tissue in PLWH display inflammatory changes, which are especially prominent when there is full-blown HALS [4]. A significant correlation between GDF15 circulating levels and pro-inflammatory cytokines was present among our patients, and GDF15 levels correlated with markers of HALS such as trunk and appendicular fat. HALS is usually associated with dyslipidemia, but there was only a weak correlation between lipid and GDF15 levels among our patients, which disappeared after age adjustment except for LDL, PUFA, and MUFA.

Dyslipidemia and HALS are contributed by the use of specific antiretroviral drugs, such as thymidine analogs and first-generation PI; others such as abacavir have been associated with an increased risk of myocardial infarction [45]. In addition, stavudine can induce inflammation at the adipose tissue level [46]. Cumulative exposure to all these drugs was highly correlated with circulating levels of GDF15, highlighting the possible contribution of some of these drugs to the metabolic and inflammatory derangements which may convey a high CVR in PLWH. It is worth mentioning that GDF15 has been recently reported to be increased in patients with genetic mitochondrial DNA diseases, including mitochondrial DNA depletion syndromes [47,48]. Thus, it cannot be ruled out that mitochondrial toxicity caused by NRTI underlies the correlation observed between GDF15 levels and treatment with thymidine NRTI. On the other hand, no studies are available to date on potential direct effects of antiretroviral drugs on cellular GDF15 secretion with the exception of a report indicating that the NNRTI efavirenz induces GDF15 expression in hepatic cells [49]. Further research will be needed to ascertain whether direct or indirect cellular actions of the distinct types of antiretroviral drugs account for the strong association between GDF15 levels and patterns of cART treatment.

Our study has the inherent limitation of being a cross-sectional study, and therefore no causal inferences can be made. However, the inclusion in our study of a remarkable number of patients spanning a wide range of cART treatment conditions and extent of HALS development allowed us to obtain substantial conclusions. Our study establishes the strong association of GDF15 levels with metabolic dysregulation and inflammation and highlights also its association with fat distribution alterations in PLWH. However, despite the existing evidence of association of circulating GDF15 levels and cardiovascular event in uninfected individuals and PLWH, we could not find such an association of GDF15 with estimated CVR in PLWH. Despite this, the association of GDF15 with insulin resistance, dyslipidemia and pro-inflammatory cytokines likely reflects an overall state of metabolic derangement and inflammation in PLWH, known to be related with vascular pathologies. In this sense, the possibility that the assessment of CVR by risk scores may underestimate the actual CVR in PLWH—as no direct imaging assessment of atherosclerotic burden was available in the patients—cannot be ruled out. Moreover, some reports indicated that CVR scores may show low sensitivity and specificity in HIV-infected men, with a systematic CVR underestimation [50,51], and therefore, they may be not totally appropriate for specific assessment of CVR in PLWH.

In conclusion, PLWH show increased levels of GDF15, this rise particularly marked when treated with cART. GDF15 levels correlated positively with parameters indicative of altered metabolism (dyslipidemia, insulin resistance, metabolic syndrome) and inflammation) but did not correlate with currently used indices of CVR (Framingham score, D:A:D score) when age-adjusted. Further research is warranted to elucidate the role and potential usefulness as a biomarker of GDF15 in relation to the distinct comorbidities occurring in PLWH.

## Figures and Tables

**Table 1:** Demographics, HIV-1 infection and antiretroviral exposure parameters.

Parameter		HIV-1-Infected Patients			*p*-Value
	Controls	Naive	HALS−	HALS+	
	(*n* = 34)	(*n* = 49)	(*n* = 43)	(*n* = 60)	
Age, years	40 (39–42)	37 (31–42)	43 (39–49)	45 (41–49)	<0.0001
Sex, male %	25 (73.5)	42 (85.7)	33 (76.7)	60 (43.6)	0.4930
Means of transmission, %					<0.0001
HTSX, %	----	15 (30.6)	15 (34.9)	23 (38.3)	
IDU, %	----	2 (4.1)	11 (25.6)	23 (38.3)	
MsM, %	----	32 (65.3)	17 (39.5)	14 (23.3)	
Years of infection	----	2 (1–3)	12 (7–17.7)	14 (10–16)	<0.0001
AIDS, %	----	4 (8.1)	13 (30.2)	29 (48.3)	<0.0001
HCV infection, %	0 (0)	6 (12.2)	14 (32.5)	27 (45.0)	0.0034
HBV infection, %	0 (0)	3 (6.1)	2 (4.6)	5 (8.3)	0.3823
CD4 count /mm^3^	----	414 (329–606)	684 (409–908)	609 (381–839)	0.0104
CD4 nadir/mm^3^	----	364 (256–490)	161 (82–409)	119 (22–361)	<0.0001
CD8 count/mm^3^	----	826 (540–1298)	936 (763–1088)	790 (599–1169)	0.7098
Serum viral load, log_10_	----	4.4 (3.6–5.4)	2.0 (1.3–2.5)	2.1 (1.3–2.9)	<0.0001
Undetectable viral load, %	----	0 (0)	35 (81.4)	48 (80.0)	<0.0001
Baseline viral load, log_10_	----	5.1 (4.2–5.5)	5.1 (4.4–5.6)	5.2 (4.0–5.6)	0.9045
**Antiretroviral drug exposure**					
NRTI, m	----	----	197 (149–236)	226 (187–265)	<0.0001
TDF in current regimen, %	----	----	25 (58.1)	29 (48.3)	0.3251
NNRTI, m	----	----	35 (2–52)	51 (26–69)	0.0019
PI, m	----	----	47 (22–69)	48 (26–75)	0.5208
PI in current regimen	----	----	25 (59.6)	15 (30.6)	0.0400
NNRTI in current regimen	----	----	23 (40.3)	34 (69.3)	

HALS = HIV/cART-associated lipodystrophy syndrome, HTSX = heterosexual, IDU = intravenous drug user, m = months, MsM = men who have sex with men, AIDS = acquired immunodeficiency syndrome, HCV = hepatitis C virus, HBV = hepatitis B virus, NRTI = nucleoside reverse transcriptase inhibitor, TDF = tenofovir disoproxil fumarate, NNRTI = non-nucleoside reverse transcriptase inhibitor, PI = protease inhibitor.

**Table 2:** Anthropometric, cardiovascular risk factors, metabolic and fat parameters, and cytokine levels.

Parameter	Controls (*n* = 34)	HIV-1-Infected Patients			*p*-Value
		Naïve (*n* = 49)	HALS− (*n* = 43)	HALS+ (*n* = 60)	
Smokers, %	2 (5.9)	26 (54.1)	28 (65.1)	26 (48.3)	<0.0001
Diabetes mellitus, %	0 (0)	2 (4.1)	0 (0)	10 (16.7)	0.0011
Metabolic syndrome, %	0 (0)	5 (10.2)	3 (6.9)	20 (33.3)	<0.0001
Systolic BP, mm Hg	118 (111–128)	120 (110–122)	120 (110–130)	127 (111–140)	0.0232
Diastolic BP, mm Hg	69 (63–77)	70 (70–80)	75 (70–80)	80 (70–81)	0.0061
Creatinine, mg/dL	0.84 (0.74–1.02)	0.95 (0.83–1.09)	0.86 (0.77–1.02)	0.89 (0.76–1.07)	0.5181
eGFR, mL/min/1.73 m^2^	105.2 (89.9–112)	100 (81.5–114)	100 (84.4–109.7)	98 (81.7–110.4)	0.4745
BMI, kg/m^2^	24.3 (23.5–26.0)	22.7 (20.8–25.5)	23.4 (21.2–26.7)	23.5 (21.7–25.8)	0.1697
Waist circumference, cm	89 (81–92)	83 (77–91)	88 (83–97)	86 (80–95)	0.1217
Waist-to-hip ratio	0.88 (0.84–0.91)	0.87 (0.82–0.94)	0.94 (0.89–0.99)	0.93 (0.91–1.01)	<0.0001
Total body fat, %	24.8 (22.2–28.1)	16.9 (14.7–24.2)	22.7 (17.5–27.0)	18.6 (14.5–23.7)	<0.0001
Trunk fat, kg	9.1 (7.8–10.8)	5.7 (3.9–9.4)	7.6 (6.3–10.5)	8.0 (5.1–12.2)	0.0064
Appendicular fat, kg	8.5 (6.5–10.3)	6.0 (3.4–7.6)	5.2 (3.3–7.9)	3.2 (2.2–5.3)	<0.0001
TAFR	1.1 (0.9–1.3)	1.1 (0.9–1.3)	1.4 (1.1–2.2)	2.3 (1.7–3.0)	<0.0001
Total cholesterol, mmol/L	5.4 (4.3–5.7)	4.0 (3.8–5.0)	4.9 (4.2–5.2)	5.1 (4.1–6.1)	0.0015
Triglycerides, mmol/L	0.83 (0.65–1.09)	1.0 (0.8–1.3)	2.0 (1.3–2.5)	2.0 (1.2–3.1)	<0.0001
HDL-c, mmol/L	1.5 (1.2–1.6)	1.0 (0.8–1.3)	1.2 (1.0–1.5)	1.2 (0.9–1.4)	<0.0001
LDL-c, mmol/L	3.3 (2.5–3.9)	2.4 (2.0–3.4)	2.7 (1.9–2.9)	2.9 (2.2–3.6)	0.0047
Apolipoprotein B, mmol/L	0.85 (0.77–1.0)	0.96 (0.87–1.19)	1.0 (0.79–1.19)	0.93 (0.82–1.25)	0.0553
Non-HDL-c, mmol/L	3.9 (2.8–4.5)	3.1 (2.4–3.9)	3.5 (3.0–4.0)	3.8 (2.9–4.9)	0.0097
Total cholesterol/HDL-c	3.4 (2.9–4.5)	4.1 (3.3–5.0)	3.7 (3.1–4.9)	4.2 (3.4–5.2)	0.0362
MUFAs, % total FA	23.9 (21.9–26.1)	26.1 (22.9–27.9)	28.1 (24.7–31.2)	28.1 (26.1–32.0)	<0.0001
PUFAs, % total FA	44.7 (42.3–46.2)	40.4 (37.7–43.1)	38.5 (33.1–40.9)	37.7 (33.9–41.3)	<0.0001
Glucose, mmol/L	4.8 (4.6–5.2)	5.0 (4.5–5.5)	5.2 (4.8–5.5)	5.5 (5.1–6.1)	<0.0001
Insulin, pmol/L	27.0 (20.0–52.5)	29.0 (20.0–53.0)	48.0 (30.0–83.7)	85.0 (56.0–135.5)	<0.0001
HOMA1-IR	0.5 (0.4–1.0)	0.5 (0.4–0.9)	0.8 (0.6–1.6)	1.6 (1.0–2.5)	<0.0001
Adiponectin, µg/mL	2.9 (1.5–12.5)	3.8 (1.9–21.6)	1.9 (1.2–3.7)	1.3 (0.8–2.8)	<0.0001
Leptin, log_10_	3.7 (3.4–3.9)	3.1 (2.8–3.4)	3.6 (3.0–3.9)	3.4 (2.9–3.8)	0.0018
C-reactive protein, mmol/L	0.9 (0.9–1.4)	2.0 (0.9–5.0)	1.5 (0.9–5.8)	2.1 (0.9–3.6)	0.0008
FGF21, µmol/L	35.4 (24.9–61.9)	57.3 (25.6–228.5)	64.4 (33.7–123.6)	93 (49.03–125)	<0.0001
Interleukin 6, pg/mL	107 (65–165)	156 (97–644)	126 (70–241)	128 (65–264)	0.0427
Interleukin 8, pg/mL	405.5 (280–550)	494 (285–719.5)	429 (295.5–658.5)	491 (339–898)	0.0636
TNF-α pg/mL	345 (257–465.8)	615 (288.3–949.5)	399 (307–527.5)	386 (252–554)	0.0010
MCP-1, pg/mL	187 (157.3–240)	220 (168.5–270.5)	196 (167–218.5)	196 (162–230)	0.1266
GDF15, ng/mL	0.31 (0.15–0.4)	0.55 (0.33–0.94)	1.03 (0.64–2.55)	1.19 (0.8–1.73)	0.0039

Parameters are expressed as median (interquartile range) unless specified. HALS = HIV-1/HAART-associated lipodystrophy syndrome, BP = blood pressure, eGFR = estimated glomerular filtration rate, BMI = body mass index, TAFR = Trunk/appendicular fat ratio, HDL-c = high density lipoprotein cholesterol, LDL-c = low density lipoprotein cholesterol, FA = fatty acids, MUFA = monounsaturated fatty acids, PUFA = polyunsaturated fatty acids, HOMA1-IR, homeostasis model assessment of insulin resistance 1, FGF21 = fibroblast growth factor 21, GDF15 = growth differentiation factor 15, TNF-α = tumor necrosis factor alpha, MCP-1 = monocyte chemoattractant protein 1.

**Table 3:** Correlations of serum GDF15 levels with anthropometric, metabolic, infectious and treatment factors in PLWH.

	Serum GDF15 (pg/L) (Crude Analysis)		Age-Adjusted Serum GDF15	
	*r*	*p*	*r*	*p*
Age	**0.453**	**<0.001**		
Fasting insulin	**0.327**	**<0.001**	**0.280**	**0.002**
HOMA1-IR	**0.326**	**<0.001**	**0.272**	**0.003**
FGF21	**0.422**	**<0.001**	**0.425**	**<0.001**
MUFAs	**0.479**	**<0.001**	**0.417**	**<0.001**
PUFAs	**−0.492**	**<0.001**	**−0.468**	**<0.001**
LDL cholesterol	**−0.212**	**0.047**	**−0.278**	**0.003**
Diastolic BP	**0.241**	**0.006**	**0.243**	**0.009**
Years of infection	**0.513**	**<0.001**	**0.459**	**<0.001**
NRTI exposure	**0.575**	**<0.001**	**0.434**	**<0.001**
NNRTI exposure	**0.151**	**0.002**	0.141	0.372
PI exposure	**0.417**	**<0.001**	**0.392**	**<0.001**
Interleukin-6	**0.1882**	**0.031**	**0.285**	**0.013**
Interleukin-8	**0.424**	**<0.001**	**0.371**	**<0.001**
TNF-α	**0.329**	**0.004**	**0.215**	**0.003**
Leptin	−0.165	0.063	**−0.203**	**0.029**
C reactive protein	**0.289**	**0.001**	**0.318**	**0.001**
Framingham CVR	**0.377**	**<0.001**	0.152	0.105

Significant correlations are highlighted in bold. *n* = 152. BP = blood pressure, LDL = low density lipoprotein, MUFA = monounsaturated fatty acids, PUFA = polyunsaturated fatty acids, HOMA1-IR, homeostasis model assessment of insulin resistance 1, FGF21 = fibroblast growth factor 21, GDF15 = growth differentiation factor 15, NRTI = nucleoside reverse transcriptase inhibitor, NNRTI = non-nucleoside reverse transcriptase inhibitor, PI = protease inhibitor, TNF-α = tumor necrosis factor alpha, CVR = cardiovascular risk.

**Table 4:** Correlations of anthropometric, metabolic, infectious and treatment factors with cardiovascular risk scores (%) in PLWH.

	10-yr. Framingham CVR				5-yr. D:A:D CVR			
	Crude Analysis		Age-Adjusted		Crude Analysis		Age-Adjusted	
	*r*	*p*	*r*	*p*	*r*	*p*	*r*	*p*
Age	**0.719**	**<0.001**			**0.671**	**<0.001**		
Systolic BP	**0.427**	**<0.001**	**0.362**	**<0.001**	0.233	0.081	0.120	0.285
Diastolic BP	**0.259**	**<0.001**	**0.234**	**0.005**	0.015	0.139	0.045	0.689
WHR	0.139	0.058	−0.005	0.949	**0.213**	**0.015**	**0.296**	**0.007**
Trunk fat	**0.190**	**0.010**	0.096	0.249	**0.230**	**0.005**	**0.226**	**0.043**
TAFR	**0.522**	**<0.001**	**0.268**	**0.001**	**0.496**	**<0.001**	**0.331**	**0.003**
HOMA1-IR	**0.296**	**<0.001**	**0.148**	**0.076**	**0.292**	**<0.001**	**0.323**	**0.003**
Triglycerides	**0.388**	**<0.001**	**0.358**	**<0.001**	**0.306**	**<0.001**	**0.419**	**<0.001**
Total cholesterol/HDL-c ratio	**0.288**	**<0.001**	**0.405**	**<0.001**	0.114	0.161	**0.289**	**0.009**
NRTI exposure	**0.346**	**<0.001**	0.024	0.779	**0.348**	**<0.001**	0.115	0.308
NNRTI exposure	**0.307**	**<0.001**	0.061	0.463	**0.252**	**0.002**	0.104	0.354
PI exposure	**0.376**	**<0.001**	**0.171**	**0.039**	**0.358**	**<0.001**	**0.293**	**0.008**
C reactive protein	**0.285**	**<0.001**	**0.160**	**0.048**	**0.207**	**0.012**	0.065	0.567
FGF21	0.096	0.252	0.014	0.854	**0.188**	**0.025**	**0.262**	**0.018**
GDF15	**0.412**	**<0.001**	0.016	0.853	0.050	0.598	0.061	0.429

Significant correlations are highlighted in bold. *n* = 152. BP= blood pressure, CVR = cardiovascular risk, WHR = waist/hip ratio, TAFR = trunk/appendicular fat ratio, HOMA1-IR = homeostasis model assessment of insulin resistance 1, HDL-c = high density lipoprotein cholesterol, NRTI = nucleoside reverse transcriptase inhibitor, NNRTI = non-nucleoside reverse transcriptase inhibitor, PI = protease inhibitor, FGF21 = fibroblast growth factor 21, GDF15 = growth differentiation factor 15.

## Data Availability

The datasets generated during and/or analyzed during the current study are available from the corresponding author on reasonable request.

## Supplementary Materials

The following supporting information can be downloaded at: https://www.mdpi.com/article/10.3390/jcm11030549/s1, Supplementary Methods; Table S1: Correlation of individual antiretroviral drug cumulative exposure with GDF15 circulating levels; Table S2: Differential analysis of GDF15 circulating levels among different comorbidities from the current cohort and CD4+ T cell count; Table S3: Correlations of serum GDF15 levels with anthropometric, metabolic, infectious and treatment factors in PLWH; Table S4: Correlations of anthropometric, metabolic, infectious and treatment factors with cardiovascular risk scores (%) in PLWH; Figure S1: Flow chart corresponding to recruitment of patients and controls.
