# Supplementary material for: Increased Circulating Levels of Growth Differentiation Factor 15 in Association with Metabolic Disorders in People Living with HIV Receiving Combined Antiretroviral Therapy
Source: J Clin Med. 2022 Jan 22;11(3):549. doi: 10.3390/jcm11030549 (PMC8836868; doi:10.3390/jcm11030549)

SUPPLEMENTARY MATERIALS

Supplementary Methods

Biochemistry laboratory measurements

All laboratory investigations were performed after a 12 h overnight fast and at least 15 minutes after the placement of a peripheral intravenous catheter. All lipid measurements were performed using a Hitachi 911 system (Roche Diagnostic Systems, Basel, Switzerland). Serum triglycerides were measured by a fully enzymatic standard method; HDL-cholesterol was measured by a direct method using polyethylene glycol-modified enzymes (PEGMEs) [52]. Low-density lipoprotein (LDL)-cholesterol was measured after ultracentrifugation according to the method recommended by the Lipid Research Clinic, but HDL-cholesterol was measured using the PEGME method instead of precipitation.

Body composition measurements, definition of HALS, metabolic syndrome and cardiovascular risk

Whole body DXA scans (Hologic QDR-4500A Hologic, Inc, 590 Lincoln St, Waltham, MA 02154, USA) were conducted by a single operator, blinded to subject status (patient of control) and to antiretroviral therapy for the patients. The operator was blinded to antiretroviral treatment. The percentage of fat at the arms, legs and central abdomen (calculated from the mass of fat versus lean and bone mass), as well as total lean body mass in kilograms, was recorded. To assess fat symmetry distribution, the trunk/limb fat ratio, obtained by dividing trunk fat by appendicular fat, was analyzed [24].

Serum fatty acid concentrations

The serum composition of fatty acids was determined using the method of Lepage and Roy [53]. Aliquots of 300 µL of serum were transferred into glass tubes for direct transesterification. 2 mL of methanol-benzene (4:1, v/v) with internal standard (heptadecanoic acid, C17:0) and 0.01 % butylhydroxytoluene, as antioxidant. Samples were vortexed at low speed while slowly adding 200 µL of acetyl chloride over a

period of 2 minutes. The tubes were tightly closed with teflon-lined caps and vortexed 30 seconds.

Samples were then heated for 60 minutes at 100ºC in a heating block and shaken continuously at 600 rpm. After the tubes had been cooled to room temperature, five milliliters of 6% (w/v) potassium carbonate were added. The samples were vortexed for 30 seconds and centrifuged at 2500 rpm for 20 minutes at 15ºC. The fatty acid methyl esters contained in the upper benzene phase were transferred to gas chromatography vials and stored at 4ºC until injection into the chromatograph.

The analysis was performed on a Varian CP-3900 gas chromatograph equipped with a flame ionization detector, using a capillary column model CP9205-VF-WAXms (Varian), 30m length x 0.25 mm internal diameter x 0.25 µm film thickness. Individual fatty acids were identified by order of elution and upon comparison with known commercially prepared fatty acid standards (GLC 566-C, Nu-Chek Prep Inc.). Fatty acid methyl ester peaks were identified by comparison of retention times of standards and quantified in comparison to known commercially prepared reference standards. The percentage of each fatty acid class was expressed as percentage of total fatty acids.

Supplemental Table S1

Correlation of individual antiretroviral drug cumulative exposure with GDF15 circulating levels

	GDF15			
	Crude analysis		Age-adjusted	
	r	P	r	P
Zidovudine, m	**0.385**	**< 0.001**	**0.379**	**< 0.001**
Stavudine, m	**0.307**	**< 0.001**	**0.262**	**0.004**
Lamivudine, m	**0.477**	**< 0.001**	**0.436**	**< 0.001**
Didanosine, m	0.197	0.025	0.159	0.088
Zalcitabine, m	0.147	0.095	0.091	0.328
Emtricitabine, m	0.115	0.195	0.080	0.393
Abacavir, m	**0.344**	**< 0.001**	**0.336**	**< 0.001**
Tenofovir, m	0.184	0.037	0.146	0.117
Efavirenz, m	0.187	0.034	0.112	0.232
Nevirapine, m	**0.351**	**< 0.001**	**0.370**	**< 0.001**
Indinavir, m	**0.376**	**< 0.001**	**0.289**	**0.001**
Saquinavir, m	0.182	0.040	0.131	0.161
Ritonavir*, m	0.118	0.184	0.079	0.401
Nelfinavir, m	0.067	0.452	0.070	0.452
Amprenavir, m	0.066	0.455	-0.010	0.907
Fosamprenavir, m	0.138	0.119	0.064	0.491
Lopinavir, m	0.142	0.108	0.138	0.141
Tipranavir, m	-0.008	0.920	---	----
Atazanavir, m	0.0373	0.675	0.070	0.454
NRTI, m	**0.542**	**< 0.001**	**0.498**	**< 0.001**
PI, m	**0.398**	**< 0.001**	**0.312**	**< 0.001**
NNRTI, m	0.278	0.014	0.157	0.175

Significant correlations are highlighted in bold. GDF-15 = growth differentiation factor 15, m = months, NRTI = nucleoside-analogue reverse transcriptase inhibitor, NNRTI = non-nucleoside-analogue reverse transcriptase inhibitor, PI = protease inhibitor, *= ritonavir always used as PI Booster at a dose from 100-200 mg/day

Supplemental Table S2

Differential analysis of GDF15 circulating levels among different comorbidities from the current cohort and CD4+ T cell count.

	**GDF15 (crude analysis *p*-value)**					**GDF15 (age-adjusted *p*-value)**				
		**PLWH**					**PLWH**			
	**Control**	**All**	**Naive**	**HALS-**	**HALS+**	**Contro l**	**All**	**Naive**	**HALS-**	**HALS+**
Smoking	0.231	0.119	0.345	0.885	0.275	0.134	0.123	0.476	0.723	0.141
Diabetes mellitus	NA	**0.001**	0.506	0.715	**0.042**	NA	**0.011**	0.352	0.288	**0.045**
Hypertension	NA	0.224	**0.048**	0.286	0.389	NA	0.368	0.068	0.253	0.392
Metabolic syndrome	NA	0.163	0.795	0.673	0.263	NA	0.243	0.880	0.504	0.249
HBV+	NA	0.588	0.902	0.571	0.922	NA	0.648	0.982	0.632	0.714
HCV+	NA	0.332	0.889	0.199	0.942	NA	0.324	0.678	0.353	0.965
CD4 (*correlation)*	NA	0.185	0.111	0.144	0.187	NA	0.313	0.090	0.258	0.192
Nadir CD4 (*correlation*)	NA	0.102	0.339	0.648	0.715	NA	0.318	0.135	0.718	0.722

Significant correlations are highlighted in bold. HBV+: Hepatitis B virus-positive; HCV+: Hepatitis C virus-positive; CD4: CD4+ T-cell count (mm^3). Differences in GDF15 levels in categorical variables was assessed with Mann-Whitney's U or Analysis of Covariance (ANCOVA) when age-corrected. For continuous variables, Pearson's correlation or age-adjusted partial correlations were used. *p*-values for the comparison or means or the correlation coefficients (*r*) are shown; $p < 0.05$ was set as the statistical significance threshold.

Supplemental Table S3

Correlations of serum GDF15 levels with anthropometric, metabolic, infectious and treatment factors in PLWH; non-statistically significant variables after age adjustment.

	**Serum GDF15 (pg/l) (crude analysis)**		**Age-adjusted serum GDF15**	
	r	***p***	***r***	***p***
BMI	0.002	0.979	-0.159	0.089
Waist circumference	0.003	0.977	-0.080	0.394
WHR	0.054	0.545	-0.024	0.802
Whole body fat percentage	-0.130	0.158	-0.117	0.204
Trunk fat	0.014	0.880	0.020	0.822
Appendicular fat	-0.137	0.125	-0.138	0.127
TAFR	0.068	0.453	0.112	0.181
eGFR	0.084	0.346	0.102	0.156
Creatinine, mg/dl	-0.103	0.248	0.175	0.180
Fasting glucose	0.105	0.237	-0.114	0.225
Triglycerides	0.189	0.032	0.168	0.072
Adiponectin	-0.126	0.158	-0.069	0.461
Total cholesterol	-0.075	0.399	-0.175	0.061
HDL cholesterol	0.043	0.409	0.067	0.561
Non-HDL cholesterol	0.070	0.433	0.062	0.490
Total cholesterol/HDL ratio	-0.103	0.249	0.039	0.658
Systolic BP	0.189	0.033	0.106	0.260
CD4 count	-0.061	0.542	-0.064	0.526
Serum viral load	-0.125	0.208	-0.115	0.243

n = 152. BMI = body mass index, BP = blood pressure, eGFR = estimated glomerular filtration rate, WHR = Waist/Hip ratio, TAFR = Trunk/appendicular fat ratio, HDL = high density lipoprotein.

Supplemental Table S4

Correlations of anthropometric, metabolic, infectious and treatment factors with cardiovascular risk scores (%) in PLWH, non-statistically significant variables after age adjustment.

	**10-yr. Framingham CVR**				**5-yr. D:A:D CVR**			
	Crude analysis		Age-adjusted		Crude analysis		Age-adjusted	
	r	***p***	***r***	***p***	***r***	***p***	***r***	***p***
Years of infection	**0.295**	**< 0.001**	0.032	0.698	**0.242**	**0.004**	0.093	0.408
Current CD4 cell count	0.048	0.554	0.096	0.250	0.022	0.785	0.005	0.962
Nadir CD4 cell count	**-0.217**	**0.007**	-0.023	0.786	**0.291**	**< 0.001**	-0.110	0.330
Current HIV-RNA	**0.134**	**0.024**	0.040	0.633	0.078	0.338	0.078	0.490
BMI	**0.187**	**0.011**	0.160	0.050	0.147	0.073	0.214	0.056
eGFR	-0.096	0.192	0.042	0.619	**0.253**	**0.002**	0.126	0.261
Waist circumference	**0.164**	**0.025**	0.056	0.502	**0.348**	**< 0.001**	**0.254**	**0.022**
Whole body fat	-0.084	0.252	-0.154	0.065	0.142	0.086	0.169	0.131
Appendicular fat	-0.060	0.413	-0.028	0.740	0.028	0.733	0.078	0.487
MUFAs	**0.306**	**< 0.001**	0.067	0.425	**0.230**	**0.005**	0.034	0.763
PUFAs	-0.075	0.307	-0.114	0.174	**0.196**	**0.017**	-0.107	0.341
LDL cholesterol	0.075	0.312	0.006	0.941	0.073	0.369	-0.044	0.699
Non-HDL cholesterol	0.118	0.110	0.020	0.812	**0.254**	**0.016**	0.201	0.072
Adiponectin	-0.012	0.869	-0.023	0.788	0.022	0.116	-0.045	0.692
Leptin	-0.051	0.489	0.007	0.933	0.050	0.607	0.085	0.450

n = 152. Significant correlations are highlighted in bold. BMI = body mass index, eGFR = estimated glomerular filtration rate, MUFA = monounsaturated fatty acids, PUFA = polyunsaturated fatty acids, LDL = low density lipoprotein.

Supplementary Figure S1

Flow chart corresponding to recruitment of patients and controls

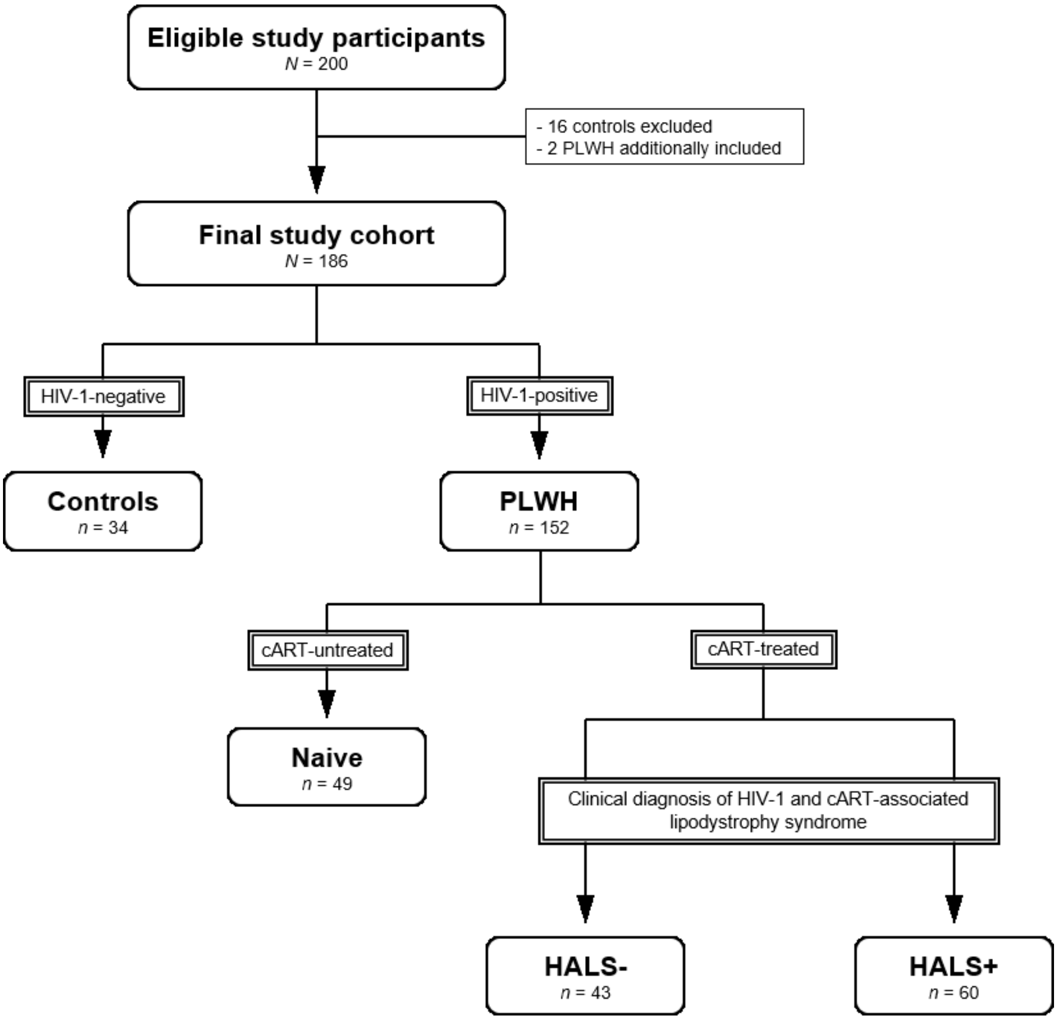

Supplement: Supplementary file 1 [file jcm-11-00549-s001.zip › jcm-1558094-supplementary.pdf]
